# The Cure of Cancer by Mattei-ism

**Published:** 1892-08

**Authors:** M. Louis Boncher


### THE CURE OF CANCER BY MATTEI-ISM.

BY

M. LOUIS BONCHER.

*Normandie Medicale* 1st April, 1892. Translated from the French, by Thomas H. Manley, M. D., New York.

It is not without painful surprise that the physician sees educated people, and often those much the best able to escape impostors, possess a strange and unaccountable faith in those medicaments, more or less marvellous, through which large industries are created and immense fortunes accumulated. Why should we spend six years of our life studying anatomy, physics, chemistry, physiology, pathology, etc., when they count for nothing in comparison with those alluring remedies which work wonderful and rapid cures in every sort of disease ?

These reflections came to mind within a few days after my opinion was asked of the cure of cancer by the method of Count Mattei. I answered that I believed myself fairly familiar with the current literature *on cancer*, but that I had never seen a case reported of a permanent cure of cancerous disease, and that I believed those cases recently reported, by this new method, of that class with which the public is familiar through the press, Nevertheless, I promised to investigate the subject fully.

From England, the country in which the new propaganda is most eagerly accepted at this moment, I procured a copy of the *Review of Reviews*, a monthly periodical which is widely circulated among English speaking people everywhere, and which, without hesitation, endeavors to popularize this mode of treatment.

I have demanded from the inventor or originator at Boulogne to send me a sample of the substances, or the recipe, that I might be the better informed of its composition. I find that it is given in granules, smaller than the *dosi-metrique*, and is designated under different heads, as anti-scrofulous, anti-cancerous, anti-febrifuge and anti-venereal. We dissolve one of these granules in a glass of water and add a teaspoonful of coffee to this solution. This should be taken in the course of the day, a tablespoonful at a time. Note well that the composition of these remedies is a secret to all but the author! Do not laugh. With these granules, are added flasks, containing electricity, red, white, yellow, blue and green. They may be given in the first, second, third or fourth dilution. This is homeopathy, pure and simple. The system is known as the electro-therapeutic.

Sufficiently edified by the notice which accompanied this invoice, I examined the various articles which appeared in the English *Review*, which had provoked such great emotion with the public by physicians occupying very high scientific positions, putting an end to the climax in publishing their own experience. This is a *résumé*.

The January article is given to the history of Mattei-ism. Lord Pajet, the English ambassador at Vienna, having been cured of a cancer, on his wife's advice, by these remedies, a journalist, pretending to have been encouraged by Dr. Kennedy, a Mattei-ist physician of London, who announced success after success, started for Boulogne, where he made a visit to Count Mattei, who was installed in a superb chateau at Rochetta, near the city. In a high tower, constructed on the model of the ancient castles, which crowned his sumptuous abode, the Count prepared his remedies. The walls were lined with fire-arms of every description, because he is haunted with a constant fear that he will be robbed of his precious secret. Here, adds the English correspondent, he aims to imitate Koch.

Finally, having at length described Boulogne and its history, he gives us the history of Mattei, who abandoned the pleasures of life and politics to study agriculture, botany and chemistry. Having his dog sick with an ulcerating mange, he followed him in his walks, noted the herbs which he bit off by the roadside and ate; he gathered them and after having distilled them, he gave the product to those having scrofula. Although, writes the correspondent, Mattei has not absolute confidence in the plants themselves, he adds a principle of electricity, something like the astral body of occultists. Does, indeed, the Black-art of the Middle Ages yet thrive in our times ? See the Sâr-Pâladan and his disciples.

In a book richly bound with gold, the gift of strangers, prominently figures the monograph of the Empress of the Austrians, who had been treated, as well as the Emperor himself, with the medicaments. How encouraging this spectacle must be to our learned confrères of Austria; to the professors, supported by the State, in the universities. This is an evidence of the confidence which is reposed in them, of the value placed on their numerous scientific researches often wrought by the sacrifice of health and fortune. Continuing the list, we find successively the testimonials of the Duke of Mecklenburg, General Ignatieff, Cardinal Lavigerie and others.

The announcement is well arranged; there is the same suggestion by the imagination. Morell Mackenzie, being interviewed, advised the erection of a small hospital which would contain five or six patients to try the method. He consented to serve as one of a committee composed of foreigners who were to examine each patient and make a report.

The article for February contains something in the way of a retraction from the Cardinal, which is in effect that the greatest virtue of the cure is in the imagination.

The issue for March contains a letter from Lawson Tait. Here are some of its fragments: "I see each week," writes this illustrious surgeon, "from eight to ten cases of cancer, the greater part of which are condemned. I could furnish almost an endless number of cases of cancer of the kidney, uterus, etc., etc., without any remedy possible. I am confident that they would resort to any treatment which would not augment their sufferings, and if but one were cured I would make it known through the medical literature of the world, letting the remedy remain a secret, and I would regard it one of the greatest blessings ever bestowed on mankind."

The articles of April and May give the constitution of the committee, composed of Mackenzie, Potter and Tait. Holy Saviour Hospital has five beds for these patients, and the representatives of Count Mattei have every liberty to ply their remedies. The patients on entrance must sign a paper that they are willing to submit to treatment. Mrs. Palmer, the directress of the hospital, has labored with the Count in Italy, but she is sceptical of anything curing cancer—the electrified granules effecting the best results in other cases.

---

MIGRAINE.—Prof. Hare recommends the following: During the intermissions, give cannabis indica in small doses (gr. $\frac{1}{4}$ of the extract), and when the patient feels that an attack is threatened give a large dose of cannabis indica (gr. j) with tincture of gelsemium gtt. xx, and the attack may usually be aborted. —*Coll. and Clin. Record.*

